# Choosing an Appropriate Infection Model to Study Quorum Sensing Inhibition in *Pseudomonas* Infections

**DOI:** 10.3390/ijms140919309

**Published:** 2013-09-23

**Authors:** Evelina Papaioannou, Putri Dwi Utari, Wim J. Quax

**Affiliations:** Department of Pharmaceutical Biology, University of Groningen, Groningen 9713AV, The Netherlands; E-Mails: evelynpapaioannou@gmail.com (E.P.); p.d.utari@rug.nl (P.D.U.)

**Keywords:** *Pseudomonas aeruginosa*, quorum sensing inhibition, animal models

## Abstract

Bacteria, although considered for decades to be antisocial organisms whose sole purpose is to find nutrients and multiply are, in fact, highly communicative organisms. Referred to as quorum sensing, cell-to-cell communication mechanisms have been adopted by bacteria in order to co-ordinate their gene expression. By behaving as a community rather than as individuals, bacteria can simultaneously switch on their virulence factor production and establish successful infections in eukaryotes. Understanding pathogen-host interactions requires the use of infection models. As the use of rodents is limited, for ethical considerations and the high costs associated with their use, alternative models based on invertebrates have been developed. Invertebrate models have the benefits of low handling costs, limited space requirements and rapid generation of results. This review presents examples of such models available for studying the pathogenicity of the Gram-negative bacterium *Pseudomonas aeruginosa*. Quorum sensing interference, known as quorum quenching, suggests a promising disease-control strategy since quorum-quenching mechanisms appear to play important roles in microbe-microbe and host-pathogen interactions. Examples of natural and synthetic quorum sensing inhibitors and their potential as antimicrobials in *Pseudomonas*-related infections are discussed in the second part of this review.

## 1. Introduction

For many years, scientists considered bacteria as autonomous unicellular organisms designed to proliferate under various conditions but with little capacity for interaction with each other and for collective response to environmental stimuli [1]. However, this view began to change around four decades ago, when emerging data provided evidence that cell-to-cell communication referred to as quorum sensing (QS) is a generic regulatory mechanism. QS enables bacteria to behave as a community and, via a density-dependent manner, launch a collective response to accomplish tasks which would be difficult, if not impossible, to achieve by an individual cell [2–4]. QS systems are widespread among many human opportunistic pathogens and are highly advantageous *in situ* where niche adaptation and symbiosis are important. Adaptation to morphological forms with better resistance to environmental threats is also aided by bacterial communication. Where establishment of successful infections is required, communication between bacteria enables them to coordinate the expression of virulence factors and overcome the defence systems of higher organisms including humans. This review discusses: (a) the QS-regulated virulence of the Gram-negative bacterium *P. aeruginosa*; (b) a number of infection models available for pathogen-host interaction studies; and (c) certain natural, and synthetic compounds, tested for their potential to rescue the infection models from *P. aeruginosa* toxicity.

## 2. Quorum Sensing in Pseudomonas aeruginosa

**Figure 1:**
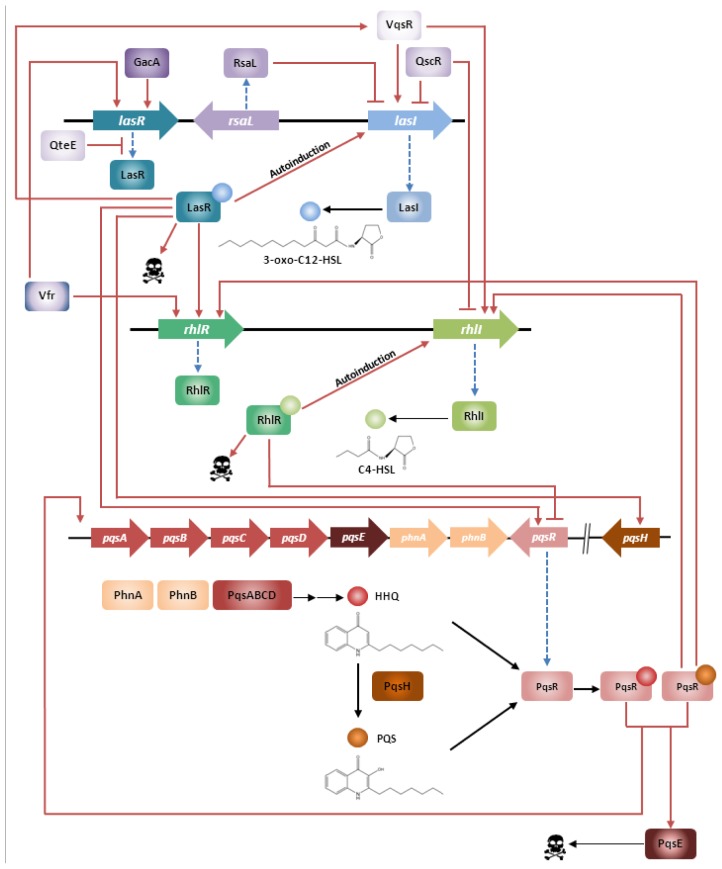
Quorum sensing (QS) in *Pseudomonas aeruginosa*. The hierarchical organisation of the two AHL-dependent QS systems in *P. aeruginosa* and its correlation with the *P. aeruginosa* quinolone signal (PQS) system is presented in the scheme below. (Skull represents virulence factor expression).

One of the most extensively studied QS systems is that of the Gram-negative opportunistic pathogen *P. aeruginosa* [5,6]. In this organism, the cell-to-cell communication is highly complex and consists of two hierarchically ordered, acyl homoserine lactone (AHL)-dependent QS systems referred to as the Las and the Rhl systems [7]. The Las system consists of the LasR transcriptional activator and of the AHL synthase LasI, which directs the synthesis of the *N*-3-oxo-dodecanoyl-homoserine lactone (3-oxo-C_12_-HSL) signal molecule [8]. Expression of a number of virulence factors including elastase, LasA protease, alkaline protease, and exotoxin A, is under the control of the Las system. Apart from its involvement in the regulation of various virulence factors, the Las system also regulates the expression of *lasI* itself, thereby creating a positive feedback loop [9] (Figure 1). By acting as an antagonist to the 3-oxo-C_12_-HSL-LasR complex, RsaL binds to *lasI* promoter, thus repressing the expression of LasI [10]. Additionally, RsaL represses production of AHL-dependent virulence factors, such as pyocyanin and cyanide [10]. LasR expression is also tightly regulated via multiple factors involving Vfr and GacA (positive feedback) or QteE (negative feedback) [11–13].

Next to its function as a signal molecule, 3-oxo-C_12_-HSL also acts as a virulence determinant in its own right by modulating the responses of the host’s defence [7]. 3-oxo-C_12_-HSL down-regulates the host defence by inhibiting activation of dendritic- and T-cells [14], promotes apoptosis of neutrophils and macrophages [15], and provokes production of inflammatory cytokines in a calcium-dependent manner [16,17].

The Rhl system consists of the transcriptional activator RhlR and the RhlI synthase which directs the synthesis of the *N*-butanoyl-homoserine lactone (C_4_-HSL) signal molecule (Figure 1). Production of rhamnolipids, including elastase, LasA protease, hydrogen cyanide, pyocyanin, the stationary-phase sigma factor RpoS, siderophores and the LecA and LecB lectins are all under the control of the Rhl system [9,18,19]. The R proteins of both systems show high fidelity for their cognate AHL and are not significantly responding to their noncognate AHL. In fact, 3-oxo-C_12_-HSL exerts only minimal RhlR activation whereas C_4_-HSL has no obvious effect on LasR. However, the Las and the Rhl systems are organized hierarchically in such a way that the Las system exerts transcriptional control over both *rhlR* and *rhlI* [7]. Despite this hierarchy, expression of *rhlI* and *rhlR* is not exclusively dependent on a functional Las system and the expression of genes such as *lecA* [20], pyocyanin, rhamnolipids and C_4_-HSL in a *lasR* mutant is delayed rather than abolished [21]. Transcriptome studies by Schuster [22] and by Wagner [23] brought to light the existence of Las- and Rhl-regulated genes and operons throughout the chromosome supporting the idea that the *P. aeruginosa* QS circuitry constitutes a global regulatory system.

The Las and the Rhl systems are further modulated by the *P. aeruginosa* quinolone signal 2-heptyl-3-hydroxy-4-quinolone (PQS) which increases the level of complexity to the QS network. PQS synthesis is controlled by both the Las and Rhl systems, whereas PQS itself controls the expression of RhlR and RhlI [24]. The PQS biosynthesis is aided by *pqsABCD* operon and regulated by the PqsR regulator, also referred to as MvfR. PqsR is a membrane-associated transcriptional activator that also regulates the production of elastase, 3-oxo-C_12_-HSL, phospholipase and pyocyanin [25]. Exogenous PQS was shown to induce expression of elastase B and of *rhlI* [6]. Aendekerk and co-workers [26] added to the understanding of PQS’s function by demonstrating that *P. aeruginosa* strains carrying mutations in the QS-regulated multi-drug efflux pump MexGHI-OpmD, that they were unable to produce wild type levels of either PQS or HSL and that these mutant strains were also unable to establish successful infections in mice and plant models. In addition, growth defects as well as altered antibiotic susceptibility profiles were observed for these strains. However, the phenotypes of these mutants could be restored to wild-type by the addition of exogenous PQS suggesting that the AHL/PQS-dependent QS-regulatory network plays a central role in coordinating virulence, antibiotic resistance and fitness in *P. aeruginosa* [26].

Since QS hierarchical order is observed in *P. aeruginosa* grown in rich medium, interesting behaviours can be seen under different growing conditions [27]. For instance, under phosphate-depletion conditions, the Las system seems to be dispensable for *rhl* and *pqs* activation. A recently published paper [28] suggested that *amb* genes in *ambBCDE* operon are responsible for the biosynthesis of 2-(2-hydroxyphenyl)-thiazole-4-carbaldehyde (IQS), a molecule important in integrating quorum sensing and stress response. This system was further modulated by phosphate signalling, particularly PhoB. Production of IQS circumvents the *las* null mutation and activates QS- and virulence-associated genes in a *las-*independent manner [28]. This finding might explain the enigmatic phenomena where the *las*-defective clinical isolate maintains persistent infection, as for example in the pulmonary infection observed in cystic fibrosis (CF) patients.

## 3. *P. aeruginosa*-Related Human Infections

*P. aeruginosa* is a ubiquitous Gram-negative pathogen adapted to a variety of niches and has been described as one of the most common causes of nosocomial infections. This pathogen is capable of infecting virtually all tissues and nearly all clinical cases of *P. aeruginosa* infections can be associated with the compromise of host defence [29]. The high-risk groups for acquiring *P. aeruginosa* infections include severely-burned, HIV- and neutropenic-patients. CF patients also suffer from chronic *P. aeruginosa* infections, and this pathogen has been described as the major cause of mortality among this group [30,31]. Despite the aggressive antibiotic intervention, currently used in an attempt to clear the *P. aeruginosa* infection from CF patients, clearance is almost impossible to achieve. As a result, the majority of patients become persistently colonized by *P. aeruginosa* that leads to eventual death. Recent studies have also demonstrated *P. aeruginosa* to be the principal etiological agent of microbial keratitis (MK) associated with contact lens wear. Around 140 million people worldwide are contact-lens users and therefore at risk of developing a *Pseudomonas*-related MK infection which in extreme cases can result in complete sight loss [32,33]. In addition, colonization by *P. aeruginosa* has been described in cases of otitis, endocarditis, as well as in cases of acute and chronic non CF-associated pulmonary infections [32]. A large variety of QS-regulated virulence factors including lipopolysaccharide, pili, proteases, exoenzymes, hydrogen cyanide, exotoxin A, and rhamnolipids are crucial for the establishment of a successful *P. aeruginosa* infection in the eukaryotic host [34].

## 4. Models Available for Studying the Pathogenicity of *P. aeruginosa*

Understanding the pathogen-host relationship at both the cellular and molecular level is essential for identification of new targets and for development of new strategies to fight infection. Hence, molecular analysis of host-pathogen interactions would benefit from the use of model systems that allow a systematic study of the factors involved. A number of evolutionary divergent hosts such as mammalian cell lines [35], amoeba [36], nematodes [37], insects [38], and rodents [39] have been chosen for studying the pathogenesis of *P. aeruginosa*. Important experimental data that contributed to the understanding of *P. aeruginosa* pathogenesis have been derived, but none of the available models resemble the pathology of the disease as experienced by humans. Bigger and more complex animals, such as the CF pig model and the rodents, may not be feasible to use in studies due to the cost, space requirements and ethical considerations (Table 1). On the other hand, invertebrate model hosts, for example nematodes, have major drawbacks such as the lack of an adaptive immunity, a true complement system and an immune cell multilineage complexity, all of which are characteristics of humans. Hereby, we described animal models utilized for studying *P. aeruginosa* pathogenesis, followed by the feasibility of studying alternative drugs, such as quorum sensing inhibitors in these models.

### 4.1. Plants

Plants, due to their Toll-like receptors, are considered excellent alternative models for studying the pathogenesis of several microbes. The plant *Arabidopsis thaliana* was used as a model to study the virulence of a *P. aeruginosa* clinical isolate and the results indicated that the pathogen employs a similar subset of virulence factors to elicit disease in plants as it does in animals [40]. *Lemna minor* (duckweed) is widely used as a plant model for studies in plant physiology, genetics, ecology and environmental monitoring [41]. The reasons that make duckweeds a preferred model for such studies are their small size, their ability to rapidly undergo vegetative reproduction thus forming genetically uniform clones, and their high sensitivity to organic and inorganic substances. In 2010, Zhang and colleagues [41] developed an experimental model system using the duckweed as a simple and convenient host allowing for large scale studies on bacterial infections [41]. The authors also demonstrated the potential of using this model system to screen antibacterial compounds by co-cultivation of duckweeds with pathogenic bacteria. A small amount of *P. aeruginosa* PAO1 suspension was enough to elicit disease symptoms to the duckweed. These symptoms included collapsing of the fronds and inhibition of both reproduction and growth at day 1 after inoculation, followed by chlorosis and complete maceration at day 2–3 post-inoculation. Bacterial biofilm formation on the roots was detectable on day 5 post-inoculation. PAO1 recombinant strains overexpressing quorum quenching enzymes (hPONs and *Bacillus* AiiA) had less profound effects on duckweed growth and displayed a reduced virulence at day 5 post-inoculation compared to the wild-type [41]. Attenuated virulence could be also observed for PAO1 QS mutant strains of Δ*rhlI*, Δ*lasI* and Δ*rhlI*/Δ*lasI*. Based on the above observations, it can be concluded that the duckweed model provides a highly sensitive and effective assay system for studying the pathogenesis of *P. aeruginosa* strains and it can possibly serve as a model system to test the functions of virulence genes of pathogenic bacteria in general [41]. Plant models face, however, limitations such as growth and size differences between individuals.

### 4.2. Cell Lines

At the cellular and molecular level, the bacterial pathogenesis mechanism and host response of cells have been studied using human cell lines. Using a microarray approach, Ichikawa *et al.* in 2000 [35] revealed alterations in gene expressions of A549 human lung carcinoma cells upon interaction with *P. aeruginosa*. An important gene encoding transcription factor IRF-1 (interferon regulatory factor 1), is upregulated in such a setting [35]. Exposure of IRF-1-deficient mice to lipopolysaccharide (LPS) and exotoxin A of *P. aeruginosa* showed reduction in production of tumor necrosis factor alpha (TNF-α) and interleukin 1 (IL-1), which indicates the importance of IRF-1 in eliminating *P. aeruginosa* infection [42]. The Caco-2 cell line (human intestinal epithelial cell) is sensitive to 3-oxo-C_12_-HSL, which causes apoptosis of the cells [16]. Transfection of PvdQ, an acylase active against 3-oxo-C_12_-HSL into these cells have proven to be effective to protect the cells from apoptosis [43]. This result indicates that quorum quenching can be a potential therapy for *P. aeruginosa* infection. A collection of numerous wild-type and *Cftr* −/− null cell lines, which can be cultured as either polarized or non-polarized, is currently available for mimicking CF infection *in vitro*. CF-lung-derived primary epithelial cells have also added significant knowledge to the cell biology of CF [44–48]. However, using cell lines has a major drawback due to the lack of differentiation in phenotypes and lack of complexity in intact organs [44].

### 4.3. *Dictyostelium discoideum*

The simplest organism that has been established as a model to study *P. aeruginosa*-host interactions is the social soil amoeba *Dictyostelium discoideum* [36,49,50]. The genome size of *D. discoideum* is about 34 Mb and the haploid nature of this genome offers *D. discoideum* a major advantage to other infection models as it allows the generation of a rich variety of mutants. *P. aeruginosa* virulence factors have been studied in *D. discoideum* by using simple plating assays comparing *P. aeruginosa* parental and mutant strains [51]. The Rhl system plays an essential role in controlling virulence of *P. aeruginosa* in *D. discoideum* infection models, since isogenic mutants deficient in Rhl system showed reduced virulence [36]. An important finding that supports the establishment of this amoeba as a pathogenesis model is the positive correlation between *D. discoideum* and a rat model infected with less virulent, MexEF-OprN-efflux pump-overproducer strain [36]. The contribution of phenazines and rhamnolipids to the killing of *D. discoideum* was examined and the results indicated that these factors are not responsible for *P. aeruginosa* virulence in *D. discoideum* [52]. Furthermore, purified pyocyanin, a phenazine that applies oxidative stress to eukaryotic cells, when added to *D. discoideum* at concentrations sufficient to kill mammalian cells, did not affect the viability of *D. discoideum*. This can be explained by the fact that *D. discoideum* is a soil organism that usually encounters a variety of different oxidative radicals. It is therefore expected that *D. discoideum* has evolved a wide range of mechanisms to deactivate such radicals [52]. Despite its many advantages as an infection model, the inability of *Dictyostelium* to survive at temperatures above 27 °C is a major drawback as *P. aeruginosa* and many other pathogens express most of their virulence traits at higher temperatures [53].

### 4.4. *Caenorhabditis elegans*

One of the simplest invertebrate models for studying *P. aeruginosa*-host interactions is the nematode *Caenorhabditis elegans* normally found in the soil. The hermaphrodite *C. elegans* which can grow up to 1 mm in length possesses several advantages as a model. Examples of such advantages are its small size, the simple conditions it requires for growth and its rapid generation time. The nematodes are routinely propagated in the laboratory on petri dishes containing lawns of the auxotroph *Escherichia coli* OP50. Regardless of its simplicity, the innate immunity pathway of this nematode shares similarity with that of mammals. Antimicrobial proteins in *C. elegans* are produced via the PMK-1 p38 mitogen-activated protein kinase (MAPK) signalling cassette, which is related to the Toll-like receptor cascade found in mammals [54]. The well-developed genetic and molecular tools, as well as the complete genome sequence being available, contribute to the advantages of using *C. elegans* as a model for studies on bacterial pathogenesis [55–57].

Depending on the experimental conditions used, in agar-based assay, *P. aeruginosa* is known to kill *C. elegans* in four distinct ways. When grown on a rich, high-osmolarity medium, this pathogen causes lethal paralysis to the nematodes via a diffusible toxin, phenazine-1-carboxylic acid, in a pH-dependent manner [58]. However, when PA14 is grown on a minimal medium, the nematodes undergo a slow infection process that lasts several days and involves accumulation of bacterial cells in the nematode’s intestines [59,60]. Yet another virulence mechanism was reported by Darby *et al.* [61]. This killing mechanism is the result of the action of hydrogen cyanide which requires the functionality of both the Las and the Rhl QS systems and causes a rapid neuromuscular paralysis [61]. In addition, “red death” of *C. elegans* occurs when the bacteria are grown in minimal medium with depletion of phosphate, where there is an activation of the phosphate signaling (PhoB)—the MvfR-PQS pathway of QS—and the pyoverdin iron acquisition system [62]. In a different setting termed as liquid killing assay, a recently discovered killing mechanism of *P. aeruginosa* is based on the siderophore pyoverdin that induces a hypoxia response and death in *C. elegans* in a liquid medium [63].

Despite its simplicity as an organism, *C. elegans* has significantly contributed to the understanding of both the pathogen’s and the host’s behaviour during the infection. It has been demonstrated that lethal paralysis of the nematodes by *P. aeruginosa* requires a functional copy of EGL-9, a protein strongly expressed in the nematode body wall and pharyngeal muscles [61]. Functional *lasR* seems to be only necessary for slow-killing and not for fast-killing of *C. elegans* by *P. aeruginosa* PA14 [56]. Even though *C. elegans* is a suitable and highly preferred model for studying the pathogenesis of *P. aeruginosa*, the nematode faces limitation in a lack of adaptive immunity.

### 4.5. *Drosophila melanogaster* (Fruit Flies)

The diptera *Drosophila melanogaster* has a long history as an animal model. Initiated by T.H. Morgan in 1910 as a model for studying heredity [64], by the year 2000 nearly 120 Mb of the fly’s genome had been successfully sequenced and annotated [65]. One of the advantages of this animal as a model for performing pathogenesis study is the shared similarity to the mammalian innate immune response. In both *D. melanogaster* and mammals, Toll family receptors signal through Rel family transactivators, mediating responses that are specific to different classes of pathogens [66]. *D. melanogaster* has an innate immune system similar to that of mammals and this makes it a favourable model for studying bacterial pathogenesis.

Even though physical barriers and antimicrobial substances protect *D. melanogaster* from microbial attacks, pathogens such as *P. aeruginosa* are capable of penetrating the exoskeleton or the intestinal epithelium and cause an infection following what is referred to as the “physiological” or “natural” route of infection [66]. *P. aeruginosa* in particular can cause infection by two ways: (a) it can be distributed in the food used to feed *D. melanogaster* larvae or adult flies; or (b) it can be used for pricking the dorsal part of the fly thorax body cavity with a sharp needle previously dipped into *P. aeruginosa* suspension. However, the latter method is invasive and faces the disadvantage of interfering to some extent with the host defence. Depending on the route of inoculation, there seems to be a difference in the *D. melanogaster* defence response [67], as well as a difference in the requirement of virulence factors for full bacterial pathogenesis. *P. aeruginosa* needs fully functional Las and Rhl QS systems in the fly feeding method, but not in the pricking method, since most of the QS mutants do not show attenuation in lethality, except for RhlI mutant [68]. It has been found that in the fly feeding method, RhlR is crucial in counteracting the host’s cellular immune response, possibly at the early stages of infection [69]. Experiments by D’Argenio and coworkers [70] showed that the mutation in *pil chp* genes, important in twitching motility, reduced *P. aeruginosa* PAO1 virulence in the fly pricking method. In addition, discovery of QscR function in LasI inhibition was aided by using this fly as an animal model for the assessment of mutants’ virulence [71].

When compared to alternative hosts for pathogen-host interaction studies, there are several practical limitations in using *D. melanogaster* as a model besides the inability of the fly to survive at 37 °C. Only a few strains of *P. aeruginosa* can infect via the “physiological route” and the administration of exact doses of either microorganisms or antimicrobial substances requires laborious techniques due to the small size of the fly.

### 4.6. *Galleria mellonella* (Wax Moth)

Larvae of the greater wax moth *Galleria mellonella* from Lepidoptera family are also used as a model to study human-pathogen interactions. Pathogenesis of numerous bacteria and yeasts are tested in this model, including *Acinetobacter baumannii* [72], *Burkholderia cepacia* complex [73], *Enterococcus faecium* [74], Enteropathogenic *Escherichia coli* [75], *Legionella pneumophila* [76], *Listeria monocytogenes* [77,78], *Pseudomonas aeruginosa* [79–83], *Staphylococcus aureus* [84], *Streptococcus pneumonia* [85], *Aspergillus flavus* [86], *Aspergillus fumigatus* [87], *Candida albicans* [88,89], *Cryptococcus neoformans* [90], and *Fusarium* spp. [91].

Compared to *D. melanogaster*, *G. mellonella* larvae are larger in size (250 mg), and therefore enable convenient injection of precise bacterial amounts into the hemocoel. In contrast to the *C. elegans* and the *Drosophila* models, a microscope is not required for the manipulation of *G. mellonella*. The ability to grow at 37 °C is another major advantage over the other invertebrates which cannot grow at this temperature [92]. Benefited by its large size, determination of bacterial LD_50_ is possible in wax moth larvae. Injection of bacterial mutants revealed the positive correlation between increased LD_50_ and reduced virulence in burned mice models [93], indicating that this animal is an excellent model for studying relevant virulence factors in mammals.

Introduction of bacterial lipopolysaccharide (LPS) to the wax moth induces expression of genes important in pathogen recognition and the ability of the host to fight the infection [94]. Interestingly, pre-exposure of the wax moth to non-lethal amounts of pathogen increases cellular and humoral response in a dose-dependent manner preparing the host to subsequent infection [87]. These data show that despite the absence of adaptive immunity, the animal is able to mount effective protection in response to prior pathogen exposure. A study conducted by Andrejko *et al.* in 2009 [83] showed that elastase B, a metalloprotease, from *P. aeruginosa* is able to degrade an antibacterial produced by *G. mellonella* during infection. This is in line with the observed role of elastase B in mammalian systems, in which the protease is able to destroy immune components, such as complements, cytokines, IgA and IgG [83]. Protease IV of *P. aeruginosa* is known to degrade apolipophorin-III (apoLp-III) from the hemocytes and fat body of *G. mellonella* [95]. Recently, *G. mellonella* larvae has been used as a model to study the efficacy of niclosamide, an antihelminthic drug, as a quorum sensing inhibitor (QSI) for *P. aeruginosa* [96]. Administration of niclosamide gave full protection to the larvae against acute *P. aeruginosa* infection [96].

### 4.7. *Bombyx mori* (Silkworm)

Studying bacterial pathogenicity and therapeutic effects of antibiotics becomes easier when a larger animal such as the silkworm, a larva of *Bombyx mori*, is used as an infection model. The body size of the instar larvae stage of the silkworm is 5 cm, which makes handling of this model easy. Injecting bacterial and drug samples into the hemolymph or the gut of the larvae is neither hard to perform nor monitor with the help of a marker [97]. The tissues responsible for drug metabolism can be isolated from the silkworm larvae allowing studies for the pharmacodynamics of certain compounds. A number of pathogenic bacteria including *P. aeruginosa* are able to kill silkworms with the 50% lethal dose of *Pseudomonas* exotoxin A being 0.14 μg·g^−1^. Moreover, GacA, which is important for full pathogenicity in burned mouse model, is also crucial in silkworm infection [98]. The silkworm model, like *C. elegans*, *D. melanogaster* and *G. mellonella*, lacks adaptive immunity, and thus, is not an ideal model for studies in which a simulation of immune responses as shown by humans is crucial [97].

### 4.8. *Danio rerio* (Zebrafish)

A model host that combines the advantages of both the invertebrate and the rodent models is *Danio rerio*, the zebrafish. It has served as a model to study infections with a number of pathogens including *Mycobacterium marinum* [99], *Salmonella enteric* serovar Typhimurium [100], *Edwardsiella tarda* [101], *S. aureus* [102], *Streptococcus iniae* [103] and *P. aeruginosa* [104]. Zebrafish is 6.4 cm in size and relatively easy to handle. The embryos/larvae can be kept in a 96-well plate during the first five days of development. A single mating of a single adult pair of fish can generate about 200 embryos. The optical transparency of the embryos further adds to the advantages of zebrafish as a host model by allowing visualization of infection progression in real time [104]. Zebrafish has an innate as well as an adaptive immunity, both of which resemble the respective immunity of mammals. For example, zebrafish expresses Toll-like receptors, proinflammatory cytokines, complement proteins, and acute-phase response proteins.

*P. aeruginosa* is able to establish lethal infections in zebrafish embryos, which are influenced by the inoculum size and by the presence of known virulence determinants such as *lasR*, *mvfR* and *pscD*, the developmental stage of the zebrafish embryos and the presence of immune cells. Clatworthy and coworkers [104] reported that a higher number of bacterial cells are required to achieve 100% lethality in embryos inoculated at 50 h post-fertilization (hpf) than in embryos inoculated at 28 hpf. These observations suggest that *P. aeruginosa* requires its full virulence arsenal in 50 hpf embryos in order to create a niche where it can survive and divide, and that 28 hpf embryos are less immunocompetent than 50 hpf embryos.

### 4.9. Rodents

Although the previously described models already gave valuable information about *P. aeruginosa* pathogenesis, further study in a complex organism is indispensable. Small mammals, especially rodents, are the preferable model to be used. With regards to QS, numerous studies have been conducted, either for understanding host-pathogen interactions or for QS inhibition.

#### 4.9.1. Cystic Fibrosis Model

*P. aeruginosa* infections are the major cause of mortality (80%) among CF patients. The respiratory tract of CF patients offers a convenient, yet challenging, environment for bacterial growth. Infection by *P. aeruginosa* in such an environment often starts by an intermittent colonization phase, and eventually develops into a chronic infection. It is of high importance to understand the pathogenesis of *P. aeruginosa* as presented in the CF lung environment. Because no natural animal models of CF exist, scientists developed a number of different CF mouse models to aid the understanding of the progression of *P. aeruginosa* infection in a CF lung. To date, transgenic mice with CF transmembrane conductance regulator (CFTR)-defects relating to CF and lung disease have been established. The currently available CF mouse models can be broadly classified into three main categories: (1) mouse models with a complete knockout of the *Cftr* gene which contain mutations that result in a complete loss of function [105]; (2) mouse models retaining the potential for reversion to wild-type, generated using an “insertional strategy” into the target gene [106]; and (3) the later-in-time generated recombinant CF mouse-models which contain clinically relevant mutations in *Cftr*, created by introducing specific human mutations, including the ΔF508 and G551D into the equivalent mouse loci. These mouse models have been extensively reviewed elsewhere [44,107,108]. Although mouse models have been widely used and have provided precious information about CF progression, two larger animal models were developed that closer resemble the disease manifestation as observed in humans. The *Cftr* knockout pig and ferret develop a CF manifestation in multiple organs including the lungs, pancreas and gastrointestinal tract [109].

The first *P. aeruginosa* chronic pulmonary infection was developed in rats, using bacteria-containing agarose beads, introduced via the intratracheal route [110]. In 1987, Starke *et al.* [111] adapted the *P. aeruginosa*-embedded agar beads method to develop infection in mice, and they showed similar histopathological effects as in larger animals, such as rats, guinea pigs and cats [111,112]. Replacement of agarose with seaweed alginate has been performed in a chronic bronchopulmonary infection model developed by Pedersen *et al.* [113]. Agarose, agar or seaweed alginate act as artificial biofilms, retaining the bacteria in the airways and protecting them from mechanical clearing [114]. It is also possible to use a *mucA* mutant, making artificially embedded material redundant, since this isolate shows a hyperproduction of alginate [115].

Using *Cftr* −/− mouse model, Hoffman *et al.* [116] examined the effect of azithromycin towards chronic lung *P. aeruginosa* infection. Azithromycin is known to be active against Gram-positive bacteria, and a study revealed that the sub-MIC concentration of azithromycin is capable of inhibiting QS-regulated virulence factors [117]. In the abovementioned study, it is proven that azithromycin also inhibits alginate production and increases bacterial susceptibility of the host’s complement system [116].

#### 4.9.2. Burn Wound Model

Patients with burn wounds are at great risk of acquiring bacterial infections from pathogens such as *P. aeruginosa*. Local colonization of this bacterium may develop into systemic sepsis, which is often associated with a high degree of mortality. In order to understand the pathophysiology of the bacterial infection, *P. aeruginosa*-infected burned mouse models have been developed [118–122]. Topical inoculation of *P. aeruginosa* PAO1 in the burn wound induced sepsis shock indicated by elevated levels of pro-inflammatory and anti-inflammatory cytokines [119]. The importance of QS in *P. aeruginosa* pathogenicity in burn wounds was confirmed by the virulence attenuation of mutations in *lasI*, *lasR*, *rhlI*, *lasI*/*rhlI* [118] and *gacA* genes [121]. The inability of *lasR* and *lasI*/*rhlI* mutants to spread within the wound and cause septicemia is possibly caused by the low level of QS associated-virulence other than *lasA*, *lasB*, *toxA* and *rpoS* [118]. High incidence of bacterial infection in burn wounds, along with the increasing rate of antibiotic-resistance strains, makes the finding of novel antibacterials a crucial necessity. However, the main obstacle in studying potential antimicrobials is that these animal models often develop complications which are burn wound related, rather than caused by bacterial pathogenesis [120]. One of the well-known QSI, garlic extract, showed an *in vitro* inhibition of biofilm formation in burn wound isolates of Gram-negative bacteria, including *P. aeruginosa* [123]. *In vivo* examination of garlic, especially in ointment dose form, will be beneficial for its development as an antivirulence substrate.

#### 4.9.3. Foreign Body Implants Model

Another group of patients at great risk of *P. aeruginosa*-related infections are those who make use of prosthetic indwelling devices such as catheters, tracheostomy tube, and cardiac pacemakers [124]. Once the biofilm-forming bacteria, such as *P. aeruginosa*, colonize the foreign bodies, removal of the implant is usually the only alternative, since the biofilm is almost impossible to eradicate [125,126]. The protocol for *in vivo* study of antibacterial candidates using intraperitoneal foreign-body infection in a mouse model has been established [125–127]. In these studies, application of QSI (furanone C-30, ajoene or horseradish extract) solely increased bacterial clearance by the host innate immune system as compared to placebo treatment. Interestingly, synergistic antimicrobial efficacy was observed in administration of QSI in combination with tobramycin [126]. Bacterial biofilm is a compact structure, tolerant to antibiotics and to the bactericidal activity of polymorphonuclear cells (PMNs) [128]. QSI not only negatively influences biofilm formation, but also disrupts production of QS-mediated virulent factors, including rhamnolipids which act as a “shield” against PMNs. Therefore, with QSI-treatment, the biofilm is more susceptible to tobramycin and, at the same time, PMNs are more active against the pathogen [125,126,128].

#### 4.9.4. Urinary Tract Infections (UTIs) Model

*P. aeruginosa* is a common pathogen found in nosocomial catheter-associated urinary tract infections (UTIs). The tendency of this pathogen to form biofilms often leads to chronicity and recurrence of the infection [129]. A significant antibiotic resistance is rarely found in UTI cases, possibly because the urine sample used in *in vitro* assay only represents planktonic populations, without considering phenotypically distinct bacteria within biofilms [130]. Oral administration of garlic extract as a prophylactic treatment to mouse models prior to renal *P. aeruginosa* biofilm challenge significantly reduced bacterial colonization [129]. Azithromycin treatment either orally or intravenously to *P. aeruginosa*-associated UTI mouse model resulted in bacterial clearance [131]. This effect might be attributed to the inability of the bacteria to form mature biofilms in the presence of azithromycin [131].

## 5. Quorum Quenching

Scientists are focussing on three main approaches by which one could interfere with bacterial QS: (a) interfering with signal generation; (b) preventing signal accumulation; and (c) prohibiting signal reception (for a review, see references [132–134]). In this review, we discuss the current findings of QSIs using the above-mentioned interference methods in the three QS systems of *P. aeruginosa*.

A great amount of work has been performed in order to elaborate the complex QS system of *P. aeruginosa*. The gained knowledge leads to the possibility of finding alternative targets, such as pathway inhibition, for the development of novel therapies. It is suggested that the acyl group of the HSL of *P. aeruginosa* is provided by the fatty acid biosynthesis (Fab) pathway, consisting of a set of Fab proteins. Interference of this pathway, as performed by triclosan in inhibiting FabI to complete the fatty acid elongation, resulted in less production of both 3-oxo-C_12_-HSL and C_4_-HSL [135]. Yet another strategy was implemented to provide analogues of precursors important in *P. aeruginosa* signal generation. In a multistep reaction, condensation of anthranilate and β-keto-decanoic acid resulted in formation of PQS molecules. Addition of methyl anthranilate, as an analogue of anthranilate, inhibited the production of both PQS and elastase without affecting the bacterial growth [136]. Lesic and colleagues [137] introduced further potent analogues of anthranilate, termed as 6FABA, 6CABA, and 4CABA. These compounds inhibit the production of 4-hydroxy-2-alkylquinolines (HAQs), the intermediate in PQS production, and are proven to be effective in hampering *P. aeruginosa* infection in burned mouse models [137].

After the signal molecules have been synthesized and secreted into the extracellular medium, it is possible to interfere with their accumulation either by completely degrading or by inactivating them. Two types of enzymes able to degrade the AHL signal molecules have been described up to this date: (a) AHL-lactonases and (b) AHL-acylases. The least studied quorum quenching enzyme, the oxireductase, belongs to a class of enzymes capable of inactivating AHL without degradation, but via modification. Thus, these enzymes limit the amount of bioactive AHL present in the environment. AHL-lactonases hydrolyse the lactone ring in the homoserine moiety of AHLs without affecting the structure of the signal molecule any further. One of the first described and extensively studied AHL-lactonases is the AiiA produced by the Gram-positive *Bacillus* sp. 240B1 [138]. AiiA, when expressed in *P. aeruginosa* PAO1, resulted in a decrease of elastase, rhamnolipids, pyocyanin and cyanide production levels [139]. Oral administration of AiiA via food supplementation showed a reduction of virulence, since *Aeromonas hydrophila* infection in zebrafish was attenuated [140]. Despite the promising outcomes of signal degradation by AHL-lactonases, an important drawback of this approach is presented by the reversibility of the lactonolysis reaction at acidic pHs, regardless of the method used to open the lactone ring [141].

The second class of enzymes, known as AHL-acylases, is also employed by bacteria for the degradation of the AHL signal molecules. The bacterium *Variovorax paradoxus* VAI-C was the first host in which an AHL-acylase enzyme was detected [142]. Today, at least five AHL-acylases produced by a diverse range of bacteria have been extensively studied and shown to attenuate virulence in *P. aeruginosa*. These are the AiiD from *Ralstonia eutropha* [143], the AhlM from *Streptomyces* sp. [144], and the PvdQ, QuiP and HacB produced by *P. aeruginosa* PAO1 [145,146]. All of the above mentioned enzymes hydrolyse the amide bond between the acyl chain and the homoserine lactone in the AHL molecule, thus generating the corresponding free fatty acid and the homoserine lactone.

In 2003, the AiiD acylase from *Ralstonia* was described and when expressed in *P. aeruginosa* PAO1 it exhibited profound effects on the pathogen’s virulence [143]. In the presence of AiiD HSL-acylase, the swarming ability of *P. aeruginosa* was reduced. In addition, there was a significant reduction in the production of virulence factors such as elastase and pyocyanin [143]. The most important outcome of the study performed by Lin *et al.* [143] was the demonstration that AiiD can attenuate the virulence of *P. aeruginosa* in the *C. elegans* infection model where it was shown to rescue the nematodes from lethal paralysis. The latter observation hints on the possibility that this type of enzymes might be, in the future, effectively used as antimicrobial therapeutic agents.

One of the AHL-acylases in *P. aeruginosa*, PvdQ, has been studied in depth. It has been previously reported that the *in vitro* substrate specificity of purified PvdQ includes AHLs with side chains ranging in length from 11 to 14 carbon atoms [147]. The specificity of the PvdQ does not seem to be influenced by the substituent at the 3′ position of the *N*-linked acyl side chain [148]. In the presence of purified PvdQ, the accumulation levels of the 3-oxo-C_12_-HSL in growing *P. aeruginosa* cultures are severely reduced and the degradation of 3-oxo-C_12_-HSL by the protein leads to a delay in the production of PQS. Production and accumulation of C_4_-HSL are not influenced by the presence of PvdQ, confirming the inactivity of the acylase towards AHLs with short side chains. Extracellular addition of the protein and intracellular production of the enzyme produced the same results. In both cases, the production levels of the virulence factors elastase and pyocyanin significantly dropped [147]. Since both expression of PvdQ in the bacterium and administration of the protein to growing *P. aeruginosa* cultures resulted in quorum quenching *in vitro*, the nematode *C. elegans* was used as an infection model to monitor the pathogenicity of *P. aeruginosa* [149]. It has been reported that PvdQ, when overproduced in the bacterium, rescues more than 70% of the nematodes from lethal paralysis. Under different conditions which result in the slow death of the nematodes by bacterial accumulation in their gut, morphological appearance examination indicated that the development of disease-like symptoms is occurring at a slower rate when PvdQ is overproduced [149]. The importance of this AHL-acylase in interfering with QS, and in subsequently attenuating the virulence of *P. aeruginosa,* is further supported by data showing that deletion of *pvdQ* leads to higher bacterial toxicity [149].

The effect of the oxireductase enzyme has been studied by Bijtenhoorn *et al.* [150]. Oxireductase is a NADP-dependent reductase isolated from soil metagenome and designated as Bpi09. It was suggested that this enzyme might not only reduce 3-oxo-C_12_-HSL, but also 3-oxo-acyl-ACP, thus interfering with the synthesis of the signal molecule itself. Expression of Bpi09 in *P. aeruginosa* PAO1 leads to the reduction of pyocyanin production, as well as in decreased motility and poor biofilm formation. Furthermore, *in vivo* analysis using *C. elegans* paralysis assay, reveals the ability of Bpi09 to suppress virulence production in *P. aeruginosa* [150].

Enzymatic degradation of PQS signal molecule is also feasible, by the 3-Hydroxy-2-methyl-4(1H)-quinolone 2,4-dioxygenase (Hod) of *Arthrobacter nitroguajacolicus* strain Rü61 [151]. This enzyme catalyses the cleavage of PQS into carbon monoxide and *N*-octanoylanthranilic acid. Although purified Hod is shown to be active in the inhibition of PQS-signalling *in vitro*, the efficiency of the enzyme is reduced by the presence of exoprotease in the culture supernatant. PQS inactivation via *pqsA* mutation or addition of purified Hod showed virulence attenuation *in planta* using lettuce leafs [151].

The third approach of interfering with bacterial QS is by blocking the binding of the signal to the receptor, or by destroying the receptor protein. QSIs in this group are the ones that compete with AHLs for the receptor-binding site and must meet certain requirements. First, the QSI must be a molecule of a low-molecular-mass and second, it must be able to significantly reduce the expression of genes that are under QS control [141]. Equally important is the necessity for the QSI to withstand a possible clearance or degradation by the host, as well as the necessity for this molecule to be nontoxic to the infected host. Many attempts have been made to identify possible QSIs. Over the years, a number of synthetic as well as natural compounds have been identified as potential candidates and have been screened for their ability to reduce the pathogen’s virulence. The positive correlation of several QSIs effect in *C. elegans* and mice model is presented in Table 2.

### 5.1. Natural QSIs

Plants including tomato, pea seedlings, garlic, chili, water lily, soybean, carrots, crown vetch, gingko biloba, horseradish, rosemary, Tasmanian blue gum, brown algae (*Ascophyllum nodosum*), Ayuverda spice clove (*Syzigium aromaticum*), *Dalbergia thiocarpa* and *Terminalia chebula*, all produce QSIs [155,160–166]. Some bioactive compounds have been characterized as responsible for the quorum sensing inhibitor properties. Examples of such compounds are ajoene (4,5,9-trithia-dodeca-1,6,11-triene 9-oxide) in garlic extract [126,156], eugenol in clove extract [165] iberin in horseradish extract [161] and ellagic acid derivatives in *Terminalia chebula* [166]. The abovementioned natural QSIs have been verified to reduce production of virulence determinants such as the rhamnolipid and pyocyanin of *P. aeruginosa in vitro*. Some of these QSIs have been tested *in vivo*, using *C. elegans*, *D. melanogaster* or even a mouse model. It has been reported by Bjarnsholt and colleagues [141] that *in vitro*, garlic-treated *P. aeruginosa* biofilms are not only susceptible to tobramycin, but also to PMNs. These observations are identical to the ones made by Rasmussen *et al.* [159] when they exposed *P. aeruginosa* biofilms to patulin derived from the fungal *Penicillium coprobium*.

Efficacy of garlic extract was not only observed in *C. elegans* [155], but also in mouse models infected with *P. aeruginosa* via different routes. Oral administration of fresh garlic extract prior to mice urinary infection results in significantly lower renal bacterial counts and in protection of the mouse kidney from tissue destruction [129]. Furthermore, the garlic treatment was clinically tested and showed a lung function improvement in CF patients. However, the sample group was too small to give a statistically significant clinical outcome [167].

### 5.2. Synthetic QSIs

Production of synthetic AHL antagonists by modifying the AHL structure has been studied. Synthesis of AHL autoinducer analogues by Persson *et al.* [168] has shown that compounds in which the C-3 carbon on the side chain is replaced with sulphur block activity of LuxR and LasR proteins. Replacement of the head part of 3-oxo-C_12_-HSL with different aromatic rings as well as modification in the tail part showed an inhibition of LasR activity [169–171]. Screening of potential QSIs from synthetic compound library reveals the presence of PD12 (tetrazole with a 12-carbon alkyl tail) and V-06-018 (phenyl ring with a 12-carbon alkyl tail) as 3-oxo-C_12_-HSL analogues [172]. Based on the design of a previously synthesized compound, named *N*-octanoyl cyclopentylamide (C_8_-CPA), Ishida *et al.* [173] successfully produced *N*-decanoyl cyclopentylamide (C_10_-CPA); a stronger QSI that interferes with expression of *P. aeruginosa* virulence factors regulated by the *las* and *rhl* quorum-sensing systems. Synthetic *S*-phenyl-l-cysteine sulfoxide and diphenyl disulfide are not only proven to inhibit *P. aeruginosa* QS *in vitro*, but also *in vivo*, as seen in the *Drosophila* infection model [174].

Owing to the presence of high-throughput screening technique, numerous QSIs from natural sources have been found and characterized. However, isolating the compounds from its endogenous source is not a practical method, especially if an endangered species is utilized. Therefore, attempting to produce synthetic compounds is a better approach. Based on the chemical structure of natural furanones, such as that of *Delisea pulchra*, a synthetic derivative referred to as furanone C-30 was generated. The classification of C-30 as a QSI was supported by a DNA microarray analysis showing that 80% of the furanone repressed genes in *P. aeruginosa* are also QS-controlled [152]. C-30 influenced the production of exoproteases and pyoverdin. Injection of 7 μM furanone C-30 every 8 h for a six-day period into *P. aeruginosa*-infected mice resulted in a ~3 logs reduction of bacterial cell numbers in the infected lung tissues [152]. In addition, a different study by Wu *et al.* [153] showed that furanone C-30, as well as the synthetic furanone C-56, significantly increased the survival time of mice with lethal *P. aeruginosa* infections, assisted bacterial clearance by the host and reduced the lung pathology. Thus, synthetic furanones appear to be promising novel antimicrobial agents. Worries about toxicity, however, remain.

Using QSI selector systems, Rasmussen *et al.* [155] identified a number of QSI compounds with structures unrelated to the signal molecules. Examples of such compounds are the 4-nitro-pyridine-*N*-oxide (4-NPO), indole, p-benzoquinone, 2,4,5-tribromoimidazole, and 3-nitrobenzene sulphone amide. Of these, 4-NPO proved to be the most effective downregulating 37% of the QS-controlled genes in *P. aeruginosa* as demonstrated by DNA microarray-based transcriptomics. A search for the specific genes affected revealed a distinct specificity for the RhlR as the target for 4-NPO [141]. In the presence of 4-NPO, the mortality of *C. elegans* fed on *P. aeruginosa* dropped to 5%. The other potent QSI found in this study [126,156] was the garlic extract, which as explained earlier, contains ajoene as its bioactive compound. Synthetic ajoene showed synergistic antimicrobial effect with tobramycin on biofilm degradation *in vitro*, and promoted clearance of *P. aeruginosa* pulmonary infection in mice as compared to the placebo-treatment group [156].

(*R*)-Bgugaine, a pyrrolidine alkaloid from *Arisarum vulgare* is proven to have antifungal and antibacterial activity. Application of synthetic norbgugaine (demethylated bgugaine) to *P. aeruginosa* showed an inhibition of QS-regulated virulence factors, including pyocyanin, rhamnolipid, LasA protease as well as a reduction of swarming motility and biofilm production [175].

Azithromycin influences, by its presence, 10.4% of the genes of the general QS regulon of *P. aeruginosa*. Nalca *et al.* [176] reported that azithromycin-treated *P. aeruginosa* cultures exhibit a reduced expression of various proteins which are required for flagellum biosynthesis. The result was a reduced flagellum-driven motility on swimming agar plates. The same group also observed that in the presence of azithromycin, *P. aeruginosa* undergoes an impaired oxidative stress response which might account for the significant reduction of *P. aeruginosa* viability after prolonged incubation with sub-MIC (minimum inhibitory concentration) of azithromycin [176]. The *in vivo* effect of azithromycin was revealed using a mouse model of chronic *P. aeruginosa* lung infection. Treatment of such mice with azithromycin significantly improved the clearance of alginate biofilms and reduced the severity of lung pathology [114]. In a one-year period, 45 CF patients with chronic *P. aeruginosa* infection received a low-dose azithromycin treatment as an integral part of their routine treatment [177]. The study resulted in an improvement of lung function and reduction of mucoid strains of *P. aeruginosa* in sputum sample. Furthermore, a pilot clinical trial of azithromycin has been carried out in randomized, intubated colonized patients, who are at risk of rhamnolipid-dependent ventilation-associated pneumonia (VAP) [178]. Reduced occurrence of VAP was observed in the azithromycin-treated group, suggesting that virulence inhibition is a promising strategy. All these observations raise the hopes that administration of azithromycin to patients suffering from *P. aeruginosa*-related infections, including CF, might help in the management and possible eradication of these infections.

## 6. Discussion

Studying human diseases requires testing of microorganisms in appropriate model systems. Traditionally, mammalian models have been the first choice for human pathogen studies, as they show similarities to human responses to infection. However, negative factors have to be taken into consideration. For instance, the large number of animals required to be sacrificed for such studies is by itself ethically questionable. In addition, the large size of animals, and thus, the space requirements associated with it, as well as the costs and the time required for the execution of experiments, resulted in the necessity for the establishment of alternative models for studying bacterial pathogenesis. To overcome these problems, laboratories have employed models such as *C. elegans*, silkworms, *D. melanogaster* and zebrafish for studying pathogen-host interactions. Using these genetically tractable models, a lot of knowledge has been gained about the mechanisms employed by *P. aeruginosa* for the establishment of infections.

The pathogenesis of *P. aeruginosa* in evolutionary distant hosts revealed conserved virulence factors required in causing an infection in multihost systems. Hence, interference of the central system controlling this virulence production, such as deactivation of QS, will lead to bacterial attenuation, which may be observed in different hosts. Hitherto, three QS circuits are studied thoroughly: the Las, the Rhl and the PQS system. Several—but not all—key proteins in each of the QS systems are shown to be indispensable in the establishment of infection of a host model, in a particular experimental setting. LasR, for example, is important in a slow-killing, and not in a fast-killing, assay of *C. elegans*. This regulator is also essential for the full virulence required for zebrafish infection. Bacterial colonization occurring in the slow-killing assay in *C. elegans* is also occurring in a similar manner in zebrafish embryo infected with *P. aeruginosa* [59,104]. Mutation of GacA, the response regulator of the GAC two-component system, leads to a reduced bacterial virulence not only in *A. thaliana*, *C. elegans*, *G. mellonella* and silkworm, but also in burned mouse model [37,93,98,121,179].

The positive correlation of the animal models’ response in these observations strengthens the credibility of non-mammalian hosts in studying bacterial pathogenesis. The important applications for these findings are that fundamental studies and drug development can be initiated from even the simplest animal models. Since high-throughput screening and whole-body infection systems are feasible in invertebrates, analysis of large libraries of drug candidates with regards to interfering with host pathogen-interaction is possible to perform [180]. Although eventual pharmacokinetics analysis in higher animal models is irreplaceable, preliminary studies in invertebrates can be an effective start.

Increasing emergence and prevalence of antibiotic-resistant bacteria makes the currently available antibiotic less effective. While the need of novel antibacterial is alarming, screening and development of the potential drug candidate is a laboriously long process, which could take 20 years before the drugs would be allowed on market. Therefore, to circumvent this issue, the study of the novel antibacterial has to be constantly performed. Targeting virulence attenuation, instead of killing the pathogen, is considered an attractive alternative, since it gives less pressure to develop resistance. Using this approach, quorum quenching in *P. aeruginosa* serves as an excellent candidate, as it has been extensively studied and also proved to be active in eradicating bacterial infection *in vivo*.

QS in *P. aeruginosa* consists of complex molecular machineries, and its activity is induced by the environmental stimulus. Hence, exposure to different conditions during development of *P. aeruginosa* infection leads to different activity levels of QS, since the importance of QS is dissimilar in each stage. QS seems dispensable in *P. aeruginosa* acute infection, where the type III secretion system which is negatively regulated by QS plays a dominant part [181]. On the other hand, QS is proven to be involved in biofilm formation, verifying its importance in the initial steps of persistent infection. Deactivation of AHL-dependent QS, either by mutation of LasRI and RhlRI or by addition of QSIs, results in a thin, flat biofilm structure of *P. aeruginosa* mutant, which is susceptible to antimicrobial challenge and phagocytosis by PMNs [128,152]. Biofilms are thought to be an ideal configuration for persistent infection, since they provide protection from both antibacterials and the host’s immune system. Within biofilm populations, diverse bacterial entities can be found due to the nutrient and oxygen gradient along the biofilm matrix.

Genotypic and phenotypic alterations occur during the progression of persistent infections such as those observed in CF patients. Mutation in the QS systems—mostly in the Las and Rhl system—are frequently found in the chronic CF isolates. Despite this, however, the presence of 3-oxo-C_12_-HSL, C_4_-HSL, PQS and QS-associated virulence factors such as rhamnolipids in the sputa sample, indicates that some QS activity still remains [30,182,183]. This phenomenon could be due to the existence of wild type bacteria within the population *or* due to the activation of a *las*-independent pathway operated under the PQS system or conceivably under a newly discovered IQS signal molecule. Although this knowledge reveals the possibility of targeting PQS for chronic infection, it was observed, *in vitro*, that reducing PQS levels leads to the increase of cytotoxin secretion by the type III secretion system. Therefore, the delivery of PQS inhibitor should be coupled with type III blocking therapy and appropriate antimicrobials [184].

In a search for compounds that can act as QSI, many candidates, natural or synthetic, have been revealed. Examples of natural QSIs include the halogenated furanones produced by *D. pulchra* and garlic extracts, which have been shown to effectively interfere with the QS-related *P. aeruginosa* pathogenicity in the *C. elegans* and mouse infection models. The availability of appropriate infection models makes screening for compounds that result in the rescue of such models from threatening *P. aeruginosa* infections possible. However, the goal of finding an infection model that will be able to simulate bacterial infection precisely as seen in humans is yet to be reached. In addition, despite the successful application of a number of QSI in such models, their use in humans must be judged with care. Even though garlic-treatment has been successful in clearing *P. aeruginosa* infections in *C. elegans* and mice, it is far from becoming an easy treatment in clinical cases of *P. aeruginosa* infections acquired by humans. The low level of active compound in garlic requires a person to take 50 garlic bulbs a day to achieve an effective treatment, and as a result, patients tend to withdraw from such treatment. Although ajoene, the bioactive compound in garlic, has been successfully synthesized and proven to be efficacious in eliminating *P. aeruginosa* infection *in vitro* and *in vivo*, it must be first proven to be clinically safe before it can be approved as an applicable drug for humans.

## 7. Conclusions

It can therefore be concluded that generating a top model for studying host-pathogen interactions and for identifying appropriate QSIs of clinical importance is challenging and has yet not been reached. Modulating QSIs to aid their successful application in clinical treatment of *P. aeruginosa*-infected patients and finding a model in which the outcomes of testing such compounds can be directly correlated to humans remain important topics at which further research efforts should be directed.

## Figures and Tables

**Table 1:** Comparison of the infection models available for *Pseudomonas* pathogenicity studies.

Parameters	*D. discoideum*	*C. elegans*	*D. melanogaster*	*G. mellonella*	Silkworm	Zebrafish	Rodents
Size	2–4 mm	1 mm	2.5 mm	2 cm	5 cm	6.4 cm	10 cm
Generation time	12 h	4 days	10 days	30 days	40–60 days	3–4 months	10 weeks
Ease of handling	very easy	very easy	very easy	easy	easy	easy	difficult
Costs	low	low	low	low	low	low	high
Space requirements	minor	minor	minor	minor	minor	minor	major
High throughput	yes	yes	yes	yes	yes	yes	no
Speed of outcome	days	days	days	days	days	days	months
Temperature	21–25 °C	15–25 °C	18–29 °C	25–37 °C	27 °C	29 °C	37 °C
Innate immunity	yes	yes	yes	yes	yes	yes	yes
Adaptive immunity	no	no	no	no	no	yes	yes
Biological relevance	potential	potential	potential	potential	potential	confirmed	confirmed
Ethical considerations	no	no	no	no	no	yes	yes

**Table 2:** Natural and synthetic quorum sensing inhibitors tested in *C. elegans* and mouse-models. (N.D.): Not defined.

QSIs	*C. elegans*	Mouse-models	References
AHL-acylases	+	N.D.	[143,149]
Furanones	+	+	[125,126,152–154]
Garlic	+	+	[126,129,155–157]
4-NPO	+	N.D.	[155]
Azithromycin	N.D.	+	[116,131,158]
Patulin	N.D.	+	[159]
